# Effect of Direct and Indirect Materials on Stress Distribution in Class II MOD Restorations: A 3D-Finite Element Analysis Study

**DOI:** 10.1155/2020/7435054

**Published:** 2020-12-15

**Authors:** Şemsi Alp, Laden Gulec Alagoz, Nuran Ulusoy

**Affiliations:** Department of Restorative Dentistry, Faculty of Dentistry, Near East University, Nicosia, Northern Cyprus, Mersin 10, Turkey

## Abstract

The aim of this study is to investigate the stress distributions of different restoration options for class II mesio-occluso-distal (MOD) cavities. A class II MOD cavity with proximal box gingival floor 1 mm below cementoenamel junction was designed in a mandibular first molar tooth model. 3D finite-element analysis (FEA) and 3D-CAD modelling were used to examine the occlusal stresses distributed to the remaining buccal enamel (RBE), remaining lingual enamel (RLE), adhesive surfaces, and restorative materials by direct and indirect materials resulting from a 600 N of static occlusal load stimulating foodstuff. von Mises (VM) and maximum principal (Pmax) stresses were evaluated for two CAD/CAM materials and three direct materials. CAD/CAM materials exerted less stress than the direct restorative materials. Significant von Mises and Pmax stress value differences were seen among all restoration models on RBE. Reducing RLE and including it into the cavity would be a more effective option for this model in this scenario. As VM and Pmax stresses of PIHC CAD/CAM material for RBE and dentin were significantly lower than other tested materials, it may be the choice of material for indirect MOD restorations.

## 1. Introduction

Functional and parafunctional forces in the mouth can cause stress on the sound teeth, on supporting bone, on soft tissues, and on adhesively restored teeth after dental therapy [1]. Determining the distribution and analysis of these stresses are of fundamental importance in the extensive research, and they can consistently contribute to reduce the risk of dental restoration failure [2]. Today, several novel therapeutic approaches and materials have been developed to maximize the protection of healthy enamel and dentine tissues after cavity preparation and restoration of decayed teeth [3]. However, the longevity of the posterior restorations is limited by many factors. Type/shape and size of the cavity, materials, patient, and dentist are some of these factors [3]. Class II mesio-occlusal-distal cavities significantly weaken the teeth, and the restorations applied to these cavities should resist the chewing forces [4, 5]. The best restorative material and restoration type for a good treatment is still being investigated.

First permanent molars are the most affected teeth with dental caries due to the time of eruption, morphology, and position of the tooth in the oral cavity [6]. Class II mesio-occlusal-distal (MOD) restorations should be strong enough to resist the chewing forces [6–9]. Studies have been carried out for the best restorative option for extensive tissue loss for the mandibular first molar. In order to replace the lost tooth structure, dentists need appropriate restorative materials [10]. Even though the ideal material does not exist yet, the restorative material should be able to replace both enamel and dentin [8]. The elastic properties of the materials should be similar to the tooth structure [11]. However, both enamel and dentin have different elastic properties [12, 13]. Hence, care must be taken while choosing the appropriate restorative material.

In class II cavities, if all margins of the cavity are in enamel, the success rate of the restoration increases; on the contrary, it decreases when the cavity margins are in dentin or cement. Nowadays, different restorative materials are used alone or together to reduce the negative effects of the stresses on the tooth. Amalgams and composites are still being used; on the other hand, there are some other new options like glass carbomer cement (GCC) and computer-aided design/manufacturing (CAD/CAM) materials [14, 15]. Nowadays, bulk-fill composite restorations are commonly used, and it is reported that there are failure risk and potential marginal damage [4]. In another study, the flowable resin composite and bulk-fill composite combination was compared with glass ionomer cement (GIC) and bulk-fill composite, and only the bulk-fill restoration showed that glass ionomer cement used as a base under bulk-fill composite was more successful than the other groups [16]. Resin composite and nonshrink glass ionomers in bilayer restorative technique combination was compared with the bulk-fill composite which pointed out that resin composite with glass ionomer combination has better resistance to residual stresses during shrinkage and loading [4].

Finite element (FE) stress analysis is a popular technique that helps to better understand the dental biomechanics of any given geometry consisting of a mesh of elements with given mechanical properties [17–20]. The FE method separates the area to be examined into small and simple elements and is based on the principle of “moving from part to whole.” Based on the state of elements connected to each other by the nodes, the deformation of the entire structure in each node, the stresses, and the resulting variables can be calculated. Since the teeth are different in shape, alveolar bone, periodontal ligament, and many related structures do not show symmetry, they have to be simulated in 3D for a reliable analysis [21].

The aim of this study was to examine the stress distribution under a simulated occlusal loading condition in tooth tissues and different restorative materials applied with different techniques on mandibular first molar tooth having a class II mesial-occlusal-distal (MOD) cavity with proximal box gingival floor within dentin, by the 3D finite element stress analysis method.

## 2. Materials and Methods

This 3-dimensional (3D) FE study was performed using Rhinoceros 4.0 3D modeling software (McNeel North America, Seattle, WA, USA), VR Mesh studio meshing software (Virtual Gird Inc., Bellevue City, WA, USA), and Algor Fempro analysis programme (ALGOR, Inc. Pittsburgh, PA, USA). A 3D finite element model of the permanent mandibular first molar was built according to the standard anatomy described in Wheeler's atlas (“Wheeler's Dental Anatomy, Physiology and Occlusion -9th Edition” n.d.). After the models were created geometrically with the VRMesh software, they were transferred to the Algor Fempro (Algor Inc., USA) software in stl format to make them ready for analysis. The stl format is of universal value for 3D modeling programs. As the coordinate information of the nodes are stored in the stl format, there is no information loss when transferring between programs. After the model is compatible with the Algor software, it is necessary to introduce the materials and the tooth structures, as well as the created model is mandible to the software. The physical properties of materials (elasticity modulus and Poisson ratio) are defined for each of the structures that make up the models.

**Figure 1:**
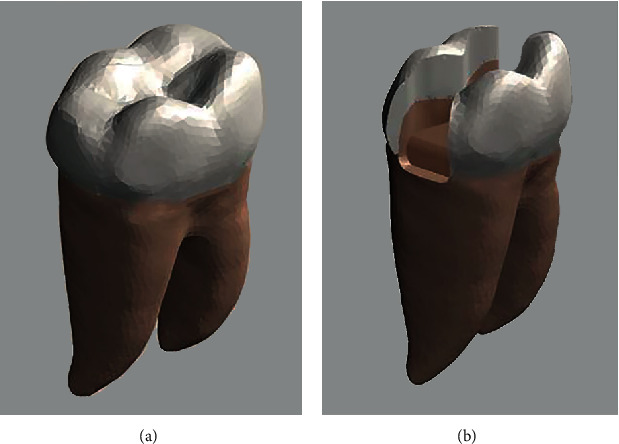
(a) Intact lower first mandibular molar model. (b) MOD cavity shape of the model.

The final modeling and scaling of the tooth were done by using the Rhinoceros software. The intact lower first molar tooth (IT) was modeled as the control group (Figure 1(a)). A thickness of 0.2 mm for periodontal ligament and 2 mm for cortical bone was modeled around the tooth [22, 23]. All oral tissues and materials used were assumed to be linear, homogenous, and isotropic.

Class II MOD cavity was modeled in Rhinoceros, and the restored models were obtained by using Boolean operations between the cavity, enamel, and dentin surfaces [16]. An MOD cavity with a proximal box gingival floor located in the dentin, 1 mm below the cement-enamel junction, was designed for the tooth. The bucco-lingual size of the cavity was 2.6 mm, the depth of the cavity was 3 mm, and the gingival wall width was 1.5 mm [24, 25]. The cavity walls were tapered with 5° from the base of the cavity to the cavosurface [12] (Figure 1(b)).

The cavity was then restored with different direct and indirect materials. The adhesive surface was modeled as 30 *μ*m [26] for the direct restorations (models 1-3), and the luting cement layer was designed as 100 *μ*m for indirect materials (models 4 and 5). Five restoration models were generated:

*Model 1 (M1)*: the cavity surface was covered with 30 *μ*m adhesive layer, and the rest of the cavity was restored with amalgam.

*Model 2 (M2)*: the cavity surface was covered with 30 *μ*m adhesive layer, and the rest of the cavity was restored with glass carbomer cement (GCC).

*Model 3 (M3)*: 1 mm resin-modified glass ionomer cement (RMGIC) base which covered the dentin floor and axial walls of the cavity was modeled. Subsequently, a 30 *μ*m adhesive layer was placed on the surface of RMGIC, and the remaining surfaces of the cavity were restored with resin composite.

*Model 4 (M4)*: after the cavity surface was covered with 100 *μ*m dual-cure resin cement layer, the rest of the cavity was restored with nanoceramic resin CAD/CAM material.

*Model 5 (M5)*: After the cavity surface was covered with 100 *μ*m dual-cure resin cement layer, the rest of the cavity was restored with polymer-infiltrated hybrid ceramic (PIHC) CAD/CAM material.

The mechanical properties of oral tissues and dental materials including Young's modulus and Poisson's ratio used for the analysis were presented in Table 1.

Bricks and tetrahedral solid elements with different numbers of elements and nodes were prepared to generate the models (Table 2).

**Figure 2:**
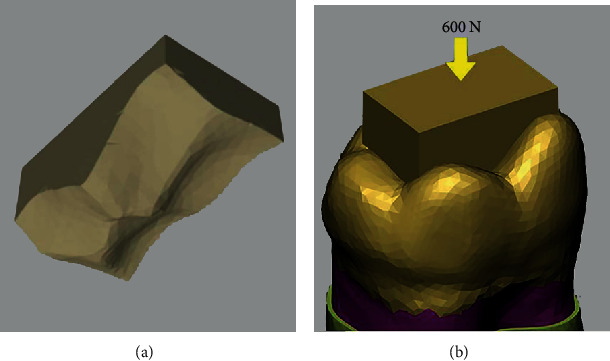
(a) Food bolus with regard to occlusal surface shape. (b) Load application on intact tooth model.

The surface of the food bolus in touch with the occlusal surface of the tooth was modeled as a copy of the occlusal tooth surface (Figure 2(a)). A 600 N static occlusal load was used parallel with the Ausiello 2017 study [3] on the food bolus (Figure 2(b)) stimulating foodstuff as in previous studies [3, 21]. von Mises and maximum principal stresses (Pmax) on the remaining buccal and lingual enamel, remaining dentin, restorative materials, and adhesive surfaces were evaluated in megapascal (MPa) separately for all models. Stresses differing below 5% were accepted as similar.

## 3. Results

Intermediate von Mises (54.86 MPa) and Pmax (12.81 MPa) stress values were observed on the sound tooth model on the enamel. In the sound tooth model, von Mises and Pmax stresses were highly observed in the cervical region (67.95 MPa) of the enamel with 67.95 MPa and 7.28 MPa values, respectively.

**Figure 3:**
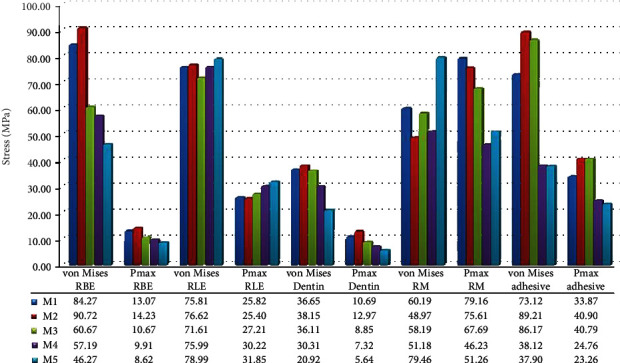
Stress values and distributions among restoration models. RBE: remaining buccal enamel; RLE: remaining lingual enamel; RM: restorative material; Pmax: maximum principal stress.

**Figure 4:**
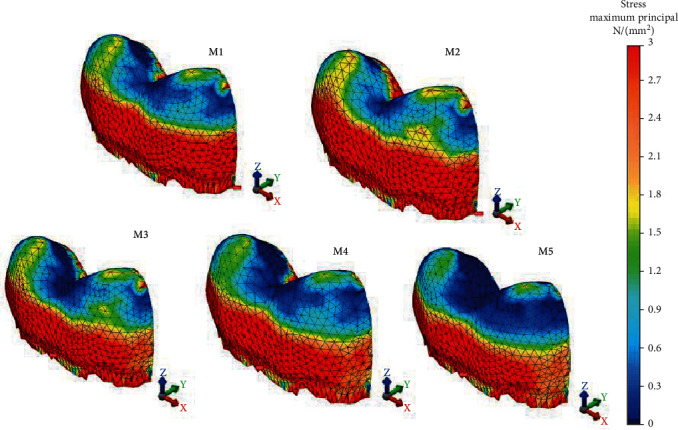
Pmax stress distributions on the remaining lingual enamel of restoration models.

The Pmax values of all restoration models and the von Mises values of M3, M4, and M5 were greater in the RLE than in RBE. M1, M2, M4, and M5 exhibited similar VM stresses to RLE, whereas M3 transferred the least stress (Figure 3). Consideration of the Pmax stress values accumulating on RLE pointed out that the comparison of all tested restoration models except M1 and M2 (25.82 MPa, 25.40 MPa) had significant differences. The two models also exhibited the lowest stress values accumulated on RLE while the maximum value accumulated on M5 with 31.84 MPa. In case of lingual enamel tissue, stress location for VM stress occurred over a narrow area in the lingual cervical region in all the models tested. On the contrary, Pmax stress was observed intensely as a large band below the mesiolingual and distolingual cusps to the lingual cervical region in all the direct and indirect restorations (Figure 4).

**Figure 5:**
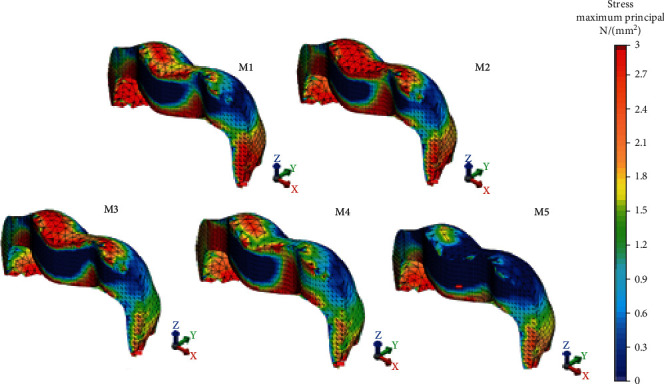
Pmax stress distributions on the remaining buccal enamel of restoration models.

Significant von Mises and Pmax stress value differences were seen among all restoration models on RBE. The minimum values were observed in M5 followed by M4, M3, M1, and M2 as seen in Figure 3. 600 N occlusal load extremely increased the stress magnitudes of glass carbomer cement on the remaining dental tissues. In terms of stress location, Pmax stress occurred mostly in the mesiobuccal cusp for M1, M2, M3, and M4 and in all the inner surface and proximal walls of the buccal wall (Figure 5). As for polymer-infiltrated hybrid ceramic CAD/CAM inlay, Pmax stress did not occur in the buccal cusps but at the proximal walls. For VM stress, the lowest value was exhibited by polymer-infiltrated hybrid ceramic CAD/CAM inlay (46.27 MPa), and it occurred in the mesio- and distobuccal cervical region of the model showing similarity with the other tested models in location.

In case of dentin, minimum von Mises and Pmax stress values were seen on indirect restoration models (M5 followed by M4), and maximum values were observed on M2 among the tested restoration models (Figure 3). Intact tooth model showed lower von Mises stress (16.92 MPa) value than restoration models and intermediate Pmax stress value (7.27 MPa). The stress distribution patterns of M1, M2, M3, and M4 were similar intensely accumulating on the remaining coronal dentin. However, for M5 and IT, the maximum stress accumulated on the coronal part of the root.

**Figure 6:**
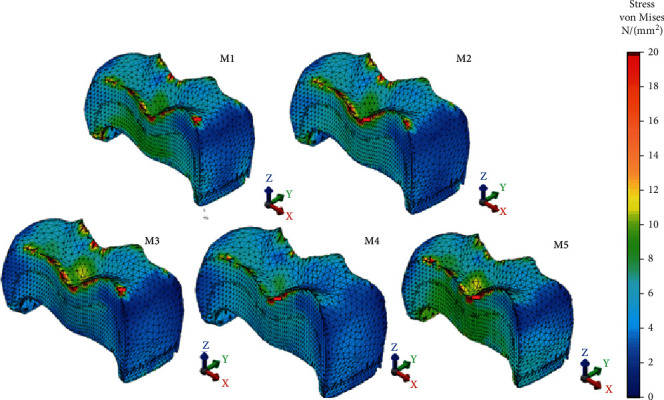
von Mises stress accumulation of restorative materials.

**Figure 7:**
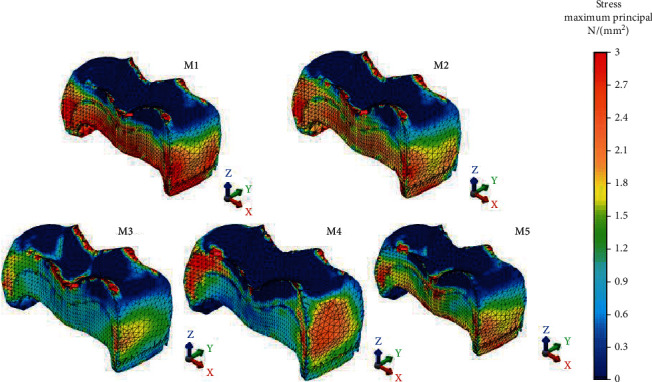
Pmax stress accumulation of restorative materials.

Maximum von Mises stress accumulation (79.46 MPa) occurred in PIHC CAD/CAM material and maximum Pmax stresses accumulated in amalgam and glass carbomer cement (79.16 MPa and 75.62 MPa), respectively. Nanoceramic resin CAD/CAM material showed the lowest von Mises and Pmax stress accumulation. Intermediate stress values occurred on resin composite. Amalgam had a similar von Mises stress value with resin composite (Figure 3). Glass carbomer had 48.97 MPa von Mises stress value, and PIHC CAD/CAM material had 51.26 MPa Pmax value. All restoration models tested showed similarities in the case of von Mises and Pmax stress distributions as seen in Figures 6 and 7, respectively.

**Figure 8:**
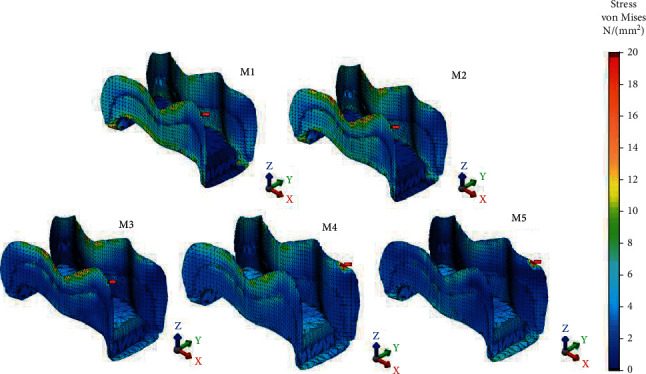
von Mises stress distributions of adhesive surfaces of restoration models.

**Figure 9:**
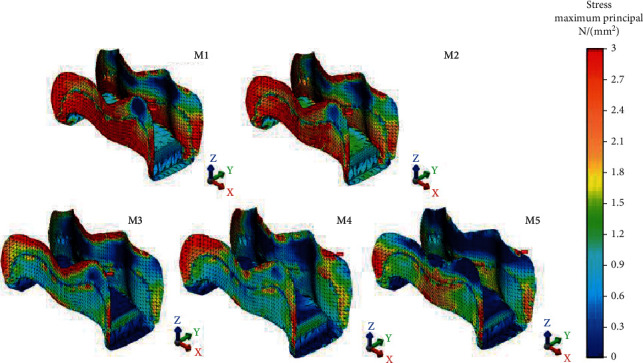
Pmax stress distributions of adhesive surfaces of restoration models.

The decreasing order of von Mises and Pmax stress values for adhesive surfaces of direct materials were as follows: M3 ~ M2 > M1 (Figure 3). Minimum von Mises stresses of bonding agents were seen on luting cement surfaces: 38.12 MPa for M4 and 37.90 MPa for M5. Pmax value of the luting cement surface of M4 (24.76 MPa) was 1.06 times higher than that of M5 (23.26 MPa). The PIHC CAD/CAM material transferred a minimum amount of stress to the adhesive, indicating that failure initiation between the luting cement/adhesive and enamel has the least likelihood. von Mises and Pmax stress distributions were presented in Figures 8 and 9, respectively.

## 4. Discussion

There are several studies investigating the occlusal stress loading of direct and indirect restorations conducted for class II MOD cavities differently restored in mandibular first molars [7, 8, 12]. Different from most previous studies, the cavity type in this study had a proximal box base 1 mm below the cementoenamel junction, in dentin [3, 17]. The aim of the present investigation was to evaluate the possible stress distribution under a simulated occlusal loading condition in the tooth structures and in the direct and indirect restoration materials when the proximal box gingival floor box was placed in dentin instead of enamel. The study intended to point out the best mechanical behavior among different restorative materials and techniques considered.

The finite element analysis (FEA) method was chosen as a tool to indicate the stress distributions in different direct and indirect adhesive materials used for class II MOD cavity restorations. The polymerization shrinkage of the resin composite, resin-modified glass ionomer cement, luting cement, and adhesives were not taken into consideration in this study. Although Ausiello et al. [27] neglected the thickness of the cement layer in their study, a 100 *μ*m-thick cement layer was simulated in our model parallel with other studies as it is clinically realistic [28, 29]. Generally, the data of finite element analysis are expressed as von Mises stress [20]. von Mises stress is a numerical stress measure combining three principal stresses (tensile, compressive, and shear) and exhibits the areas being exposed to the highest stress and consequently more prone to failure in the model [26, 30–33]. Additionally, the maximum principal stress is also accepted as a suitable index to judge the material failure that is assumed to be brittle [20]. The maximum principal stress helps us to understand the maximum tensile stress induced in the different structures, materials, and dental tissues, due to the loading conditions.

Except the von Mises stress transferred by PIHC CAD/CAM material and nanoceramic CAD/CAM material to RBE, the stress analysis of the intact tooth model showed significantly lower stress values for both enamel and dentin for all the tested restoration models. Furthermore, the minimum VM and Pmax stress values were found in the intact tooth model for RLE. On the contrary, Pmax stresses in the intact tooth were higher than the two CAD/CAM materials and resin composite for RBE but lower than those with all the tested materials for RLE. In case of dentin, except Pmax stresses of CAD/CAM materials, all the tested materials transferred higher VM and Pmax stresses when compared to the intact tooth. It was reported by previous studies that class II MOD cavities decreased the fracture resistance of intact teeth for about 59-76% [34–36]. According to an in vitro study by Reeh et al. [36], the occlusal cavity weakens the fracture resistance of the tooth by 20% and the MOD cavity weakens the fracture resistance by 63%. Our results for enamel and dentin were partly consistent with this finding. Both the stress values of PIHC CAD/CAM material and Pmax stresses transferred by nanoceramic CAD/CAM material and resin composite to RBE were lower than intact tooth. The Pmax stress of the PIHC CAD/CAM material to dentin was lower than the intact tooth, whereas the nanoceramic CAD/CAM material exhibited similar stress distribution.

Eakle et al. [37] reported that the lingual cusps of mandibular molars exhibited the highest frequency of fracture. In consistence with Eakle et al. [37], stress distribution values of buccal and lingual remaining enamel tissues in the current study showed that except von Mises stress values of glass carbomer for RBE; von Mises and Pmax stress values on RLE were higher than RBE. Regarding the stresses that occurred in RLE in the present study, in order to increase the resistance of the tooth to crown deformation, reducing the RLE and including into the cavity can be suggested in designing an MOD inlay cavity with proximal box gingival floor in dentin.

Additionally, there were significant differences between the von Mises and Pmax stress values and intact tooth. This resulted us to comment that buccal cusp reduction may also be needed in addition to lingual cusp coverage for GCC restorative material.

Although there are many materials providing many options to the clinicians, it can be challenging to choose the best one for a given situation [5]. Various materials, including amalgam, resin-based composites, ceramics, nanoceramic resin, and glass carbomers are used for posterior restorations.

There are different adhesive thicknesses in the literature: 2, 5, 10, and 30 micrometers. In the Ausiello et al. [27] study, a thin (10 micrometer) adhesive layer was used. Eliguzeloglu et al. [26] suggested that flexible materials such as glass ionomer cements, flowable composites, or nanofilled adhesives could be helpful to reduce stress under resin-based composite filling materials. In the present study, we preferred to use a 30-micrometer-thick adhesive layer to provide better flexibility. There are many investigations about the survival percentage of these types of materials for long-term evaluation [38, 39]. Opdam et al. [40] clarified that the caries risk of the patients played a significant role in the survival of these restorations. Amalgam has been considered as the primary posterior restorative material for years but it is unable to reinforce tooth structure. It is also considered as a material giving way to tooth fracture; hence, the use of this material is declining nowadays [40]. With the increasing expectation for esthetics, both dentists and patients are becoming more interested in tooth-colored materials as alternatives to amalgam in the posterior restorations [2].

Arola et al. [6] studied Pmax stress for an unrestored molar and a molar with class II MOD amalgam vs. resin composite restoration. It was found that the amount of stress between amalgam and resin composite restoration had a little difference in the magnitude of Pmax stress values. In another study, Musani and Prabhakar [1] investigated the stress distributions of resin composite and amalgam restorations for class I cavity in the mandibular first molar tooth and reported that the stress value magnitude seen on the cervical third of the crown in amalgam restoration was lower than that of resin composite. Different from the reported investigations, in this study, the differences were observed between von Mises and Pmax values on enamel and Pmax value only on dentin. Less von Mises and Pmax stresses to enamel tissues and Pmax values to dentin were transferred in the resin composite model.

Glass carbomer cement is a newly developed material containing nanosized hydroxyapatite-fluorapatite particles in powder form [41]. In a finite element study, stress accumulation on GCC and resin composite material were studied for occlusal cavities, and it was reported that GCC accumulated more stress in itself and transferred less to tooth tissue than the resin composite material [8]. The highest von Mises stress values for GCC accumulated on RBE and dentin are 90.72 MPa and 38.15 MPa, respectively, while the maximum PMax values accumulated on RBE and dentin are 14.23 MPa and 12.97 MPa, respectively. However, in this study, both stresses transferred to the remaining tissues except the Pmax value on RLE were found to be higher in GCC than in the resin composite. These results are not in agreement with the Doğan et al. study [8]. The reasons for this difference may be related to the different cavity designs used and the usage of RMGIC under the resin composite. RMGIC was used in combination with the resin composite to relocate the subgingival cavity margins allowing for a stepwise elevation of the proximal cavity floor in the present study. The RMGIC base under the resin composite restoration might have acted as a tampon layer reducing the effects of stress concentration.

With one of the newest developments in dentistry, CAD/CAM technology, it has been possible to design and fabricate restorations with mechanized, computer-aided techniques [42, 43]. With this new popular technology, new materials have been introduced in dentistry like hybrid ceramics and resin composite blocks [42, 44]. Resin composite CAD/CAM blocks consist of a polymeric matrix and dispersed fillers. Polymer-infiltrated ceramic network materials also termed as “hybrid ceramics” consist of two continuous interconnected networks (feldspathic ceramic (86% wt) and polymeric (14% wt) [44]. These materials combine the advantages of resin composites (flexibility, ease of use) and ceramics (durability, surface finish properties), while their composite-like properties make these materials easier to mill, adapt, and polish and their higher degree of polymerization strengthen the physical and mechanical properties [43]. A study held by Simsek and Derelioglu [45] in pediatric dentistry on primary maxillary molar teeth compared the fracture resistance of Vita ENAMIC CAD/CAM block (Vita Zahnfabrik, Bad Sackingen, Germany) with direct and indirect resin composite restorations for Class II cavities with gingival wall 0.5 mm above the enamel-cement junction. This investigation reported that the PIHC CAD/CAM material; Vita ENAMIC, showed greater fracture resistance than direct composite models. The results of the present study are in agreement with Simsek and Derelioglu [45] for both von Mises and Pmax stress on RBE and dentin because the von Mises stress of the PIHC CAD/CAM material on RBE was significantly lower than that of amalgam, GCC, and resin composite direct restorations. Although the VM value of PIHC CAD/CAM material was similar to that of the direct materials on RLE, its Pmax value was significantly different. However, stress values on RLE for all restoration models tested were significantly high when compared with the intact tooth model. Another study that aimed to investigate the effects of direct and indirect restorations on fracture resistance of extended buccolingual class II MOD cavities on mandibular third molars reported that Lava Ultimate CAD/CAM indirect inlays increased fracture resistance more than direct composite restorations [46]. Our results are partly in accordance with Papadopoulos et al. study [46] because the nanoceramic resin CAD/CAM material transferred lower stress to RBE and dentin than all the direct materials. However, the Pmax stress value of the nanoceramic resin CAD/CAM material to RLE was higher than all directly restored models.

Yamanel et al. [12] reported that “materials with low elastic modulus values transferred more functional stress to the tooth structures.” This statement is in accordance with our study for von Mises and Pmax stress transfers on the remaining buccal enamel and dentin. In the present study, significant differences were observed only between the M5 and M1-M4 groups indicating that PIHC CAD/CAM material with higher elasticity modulus exhibited lower VM and Pmax value on RBE. Differing from our class II MOD cavity design with gingival margins in dentin, inlay and onlay MOD restorations had gingival margins on enamel in Yamanel et al.'s study [12]. Compared to all other restoration materials, the maximum amount of von Mises stress accumulated on PIHC CAD/CAM material showing that chewing stresses are highly absorbed and least transferred to the remaining buccal enamel and dentin when ceramic materials were used. The high elastic modulus values of the PIHC CAD/CAM material accounted for this result.

On the contrary, all restoration materials with different elasticity moduli transferred high VM and Pmax stresses to RLE. Considering the class II MOD inlay cavity design with proximal gingival margins 1 mm below the CEJ in the mandibular first molar tooth, reducing the remaining lingual enamel and including it into the cavity would be a more effective option.

Ausiello et al. [27] reported that they assumed the thickness of the adhesive layers constant. However, in this study, we planned the adhesive bonding layer as 30 *μ*m and the luting cement layer as 100 *μ*m. Parallel with the Ausiello et al. [27] study since our cavity borders ended in dentin, we thought that the thickness between adhesive layers would affect the final result. Ausiello et al. [47] reported that rigid composites gave way to more cusp movements than flexible composites in class II MOD adhesive restorations. Our results are parallel with the Ausiello et al. [47] study because Vita Enamic as a PIHC material is more flexible than Lava Ultimate nanoceramic CAD/CAM material and distributed less stress in the tooth tissues.

Microleakage is still a concern for class II cavities having gingival margins in dentin. Uludag et al. [48] investigated the effects of luting cement on microleakage for the MOD cavities and found out that dual-cure resin cement (Variolink II, Ivoclar Vivadent) showed lower microleakage compared with others. For this reason, dual-cure resin cement was chosen as the luting cement material under CAD/CAM materials in the present study. The maximum amount of Pmax stress values did not exceed the tensile bond strength of the luting cement to enamel (49.3 MPa) and adhesive to enamel (42.75-65.75 MPa) figuring out that the interaction between the adhesive surfaces and dental tissues were strong [49, 50]. The comparison of Pmax stress values of adhesive surfaces showed a stunning difference among all tested models except the comparison of the GCC and resin composite model. PIHC CAD/CAM material exhibited minimum Pmax value followed by nanoceramic resin CAD/CAM material, amalgam, resin composite, and glass carbomer cement. The Pmax stress concentrations of the tested materials indicate that PIHC CAD/CAM material has the least likelihood for failure initiation between the luting cement/adhesive and enamel.

The 3D-FEA analysis is an engineering tool applied to biology, medicine, and dentistry, from orthodontics to implantology, is able to investigate the mechanical behaviour of differently structured systems in vitro by a mathematical analysis and simulation. The goal consists of creating a model as close as possible to the real one. The obtained outputs are applicable and practical, have clinical significance, and give direction to experimental and clinical research. In this study, the information obtained for suitable restorative materials for MOD cavity had gingival margins ended in dentin tissue. However, restorations have other problems such as microleakage, polymerization shrinkage of resin containing materials, and postoperative sensitivity that should be investigated. As oral conditions cannot be completely imitated by in vitro studies, further in vivo studies are needed to determine the effectiveness and durability of materials for class II MOD cavities.

## 5. Conclusion

Within the limitations of this study, the following statements can be drawn for the restoration of class II MOD cavity with proximal box gingival floor 1 mm below the CEJ in mandibular first molar:

1. As the lingual cusp of the mandibular first molar with MOD cavity was found to be more susceptible to damage than the buccal cusp, the cuspal coverage can be recommended in designing a class II MOD inlay cavity
2. Vita Enamic, the PIHC CAD/CAM material, transferred a minimum amount of stress to the adhesive materials (30 *μ*m adhesive and 100 *μ*m cement layer) and dental tissues, indicating that the failure initiation between the luting cement/adhesive and enamel has the least likelihood

For this reason, this material may be a better choice to restore class II MOD inlay cavity with proximal box gingival floor 1 mm below CEJ when it is desirable to minimize stress concentrations in the mandibular first molars.

## Figures and Tables

**Table 1:** Mechanical properties of oral tissues and dental materials including Young's modulus and Poisson's ratio used for the analysis.

Material/tissue	Young's modulus (MPa)	Poisson's ratio (*v*)	Reference
Enamel	84100	0.33	13
Dentin	18600	0.32	13
Amalgam	15000	0.30	16
Composite	10,000	0.21	17
Resin modified glass ionomer cement	12162	0.30	16
Glass carbomer cement	8300	0.3	5
Nanoceramic resin CAD/CAM material	12700	0.45	13
Polymer-infiltrated hybrid ceramic CAD/CAM material	37800	0.24	13
Dual-cure resin cement	6,000	0.30	18
Cortical bone	10700	0.3	13
Trabecular bone	1.370	0.3	19
Periodontal ligament	68.9	0.45	13
Pulp	2.0	0.45	17
Adhesive	3000	0.30	1
Food bolus	10	0.30	Ausiello 2017 (19)

**Table 2:** Number of elements and nodes of the models.

Model	Elements	Nodes
M1	285404	55677
M2	285404	55677
M3	285404	55677
M4	308036	61426
M5	308036	61426
Intact tooth	207261	40111

## Data Availability

The data used to support the findings of this study are included within the article.

## References

[1] Prabhakar A., Musani I. (2010). Biomechanical stress analysis of mandibular first permanent molar; restored with amalgam and composite resin: a computerized finite element study. *International Journal of Clinical Pediatric Dentistry*.

[2] Jiang W., Bo H., YongChun G., LongXing N. (2010). Stress distribution in molars restored with inlays or onlays with or without endodontic treatment: a three-dimensional finite element analysis. *The Journal of Prosthetic Dentistry*.

[3] Ausiello P., Ciaramella S., Martorelli M., Lanzotti A., Gloria A., Watts D. C. (2017). CAD-FE modeling and analysis of class II restorations incorporating resin- composite, glass ionomer and glass ceramic materials. *Dental Materials*.

[4] Ausiello P., Ciaramella S., Di Rienzo A., Lanzotti A., Ventre M., Watts D. C. (2019). Adhesive class I restorations in sound molar teeth incorporating combined resin-composite and glass ionomer materials: CAD-FE modeling and analysis. *Dental Materials*.

[5] Zarone F., Di Mauro M. I., Ausiello P., Ruggiero G., Sorrentino R. (2019). Current status on lithium disilicate and zirconia: a narrative review. *BMC Oral Health*.

[6] Arola D., Galles L. A., Sarubin M. F. (2001). A comparison of the mechanical behavior of posterior teeth with amalgam and composite MOD restorations. *Journal of Dentistry*.

[7] Dejak B., Mlotkowski A. (2008). Three-dimensional finite element analysis of strength and adhesion of composite resin versus ceramic inlays in molars. *The Journal of Prosthetic Dentistry*.

[8] Doğan M. S., Demirci F., Eratilla E., Eratilla V., Yavuz Y., Unal M. (2017). Evaluation of stress distribution of a new restorative material and composite resin: a finite-element analysis study. *Biotechnology and Biotechnological Equipment*.

[9] Penteado M. M., Tribst J., Dal Piva A. M. (2019). Mechanical behavior of conceptual posterior dental crowns with functional elasticity gradient. *American Journal of Dentistry*.

[10] Narayanaswamy S., Meena N., Shetty A., Kumari A., Naveen D. (2008). Finite element analysis of stress concentration in Class V restorations of four groups of restorative materials in mandibular premolar. *Journal of Conservative Dentistry*.

[11] Zafar M. S., Madinah A., Munawwarah A., Arabia S. (2014). *Zafar MS. A Comparison of Dental Restorative Materials and Mineralized Dental Tissues for Surface Nanomechanical Properties*.

[12] Yamanel K., Çaglar A., Gülsahi K., Özden U. A. (2009). Effects of different ceramic and composite materials on stress distribution in inlay and onlay cavities: 3-D finite element analysis. *Dental Materials Journal*.

[13] Dietrich T., Lösche A. C., Lösche G. M., Roulet J. F. (1999). Marginal adaptation of direct composite and sandwich restorations in Class II cavities with cervical margins in dentin. *Journal of Dentistry*.

[14] Valian A., Moravej-Salehi E., Geramy A., Faramarzi E. (2015). Effect of extension and type of composite-restored class II cavities on biomechanical properties of teeth: a three dimensional finite element analysis. *Journal of Dentistry*.

[15] Zainuddin N., Karpukhina N., Law R. V., Hill R. G. (2012). Characterisation of a remineralising Glass Carbomer^®^ ionomer cement by MAS-NMR spectroscopy. *Dental Materials*.

[16] Ausiello P., Ciaramella S., De Benedictis A., Lanzotti A., Tribst J. P. M., Watts D. C. (2020). The use of different adhesive filling material and mass combinations to restore class II cavities under loading and shrinkage effects: a 3D-FEA. *Computer Methods in Biomechanics and Biomedical Engineering*.

[17] Gloria A., Maietta S., Richetta M., Ausiello P., Martorelli M. (2019). Metal posts and the effect of material–shape combination on the mechanical behavior of endodontically treated anterior teeth. *Metals*.

[18] Tribst J. P. M., Dal Piva A. M. . O., Lo Giudice R. (2020). The influence of custom-milled framework design for an implant-supported full-arch fixed dental prosthesis: 3D-FEA sudy. *International Journal of Environmental Research and Public Health*.

[19] Trivedi S. (2014). Finite element analysis: a boon to dentistry. *Journal of Oral Biology and Craniofacial Research*.

[20] Gulec L., Ulusoy N. (2017). Effect of endocrown restorations with different CAD/CAM materials: 3D finite element and weibull analyses. *BioMed Research International*.

[21] Toksavul S., Zor M., Toman M., Güngör M. A., Nergiz I., Artunç C. (2006). Analysis of dentinal stress distribution of maxillary central incisors subjected to various post-and-core applications. *Operative Dentistry*.

[22] Fongsamootr T., Suttakul P. (2015). Effect of periodontal ligament on stress distribution and displacement of tooth and bone structure using finite element simulation. *Engineering Journal*.

[23] Sugiura T., Yamamoto K., Horita S., Murakami K., Tsutsumi S., Kirita T. (2016). The effects of bone density and crestal cortical bone thickness on micromotion and peri-implant bone strain distribution in an immediately loaded implant: a nonlinear finite element analysis. *Journal of Periodontal & Implant Science*.

[24] Alshiddi I. F., Aljinbaz A. (2016). Fracture resistance of endodontically treated teeth restored with indirect composite inlay and onlay restorations - An _in vitro_ study. *The Saudi Dental Journal*.

[25] Forster A., Braunitzer G., Tóth M., Szabó B. P., Fráter M. (2019). In vitro fracture resistance of adhesively restored molar teeth with different MOD cavity dimensions. *Journal of Prosthodontics*.

[26] Eliguzeloglu E., Eraslan O., Omurlu H., Eskitascıoglu G., Belli S. (2019). Effect of hybrid layer and thickness on stress distribution of cervical wedge-shaped restorations. *European Journal of Dentistry*.

[27] Ausiello P., Franciosa P., Martorelli M., Watts D. C. (2011). Numerical fatigue 3D-FE modeling of indirect composite-restored posterior teeth. *Dental Materials*.

[28] Sangeetha A., Padmanabhan T., Subramaniam R., Ramkumar V. (2012). Finite element analysis of stresses in fixed prosthesis and cement layer using a three-dimensional model. *Journal of Pharmacy and Bioallied Sciences*.

[29] Li Z. C., White S. N. (1999). Mechanical properties of dental luting cements. *The Journal of Prosthetic Dentistry*.

[30] Maravić T., Vasiljević D., Kantardžić I., Lainović T., Lužanin O., Blažić L. (2018). Influence of restorative procedures on endodontically treated premolars: finite element analysis of a CT-scan based three-dimensional model. *Dental Materials Journal*.

[31] Pegoretti A., Fambri L., Zappini G., Bianchetti M. (2002). Finite element analysis of a glass fibre reinforced composite endodontic post. *Biomaterials*.

[32] Pierrisnard L., Bohin F., Renault P., Barquins M. (2002). Corono-radicular reconstruction of pulpless teeth: a mechanical study using finite element analysis. *The Journal of Prosthetic Dentistry*.

[33] Eraslan Ö., Eraslan O., Eskitaşcioǧlu G., Belli S. (2011). Conservative restoration of severely damaged endodontically treated premolar teeth: a FEM study. *Clinical Oral Investigations*.

[34] St-Georges A. J., Sturdevant J. R., Swift E. J., Thompson J. Y. (2003). Fracture resistance of prepared teeth restored with bonded inlay restorations. *The Journal of Prosthetic Dentistry*.

[35] Dalpino P. H., Francischone C. E., Ishikiriama A., Franco E. B. (2002). Fracture resistance of teeth directly and indirectly restored with composite resin and indirectly restored with ceramic materials. *American Journal of Dentistry*.

[36] Reeh E. S., Messer H. H., Douglas W. H. (1989). Reduction in tooth stiffness as a result of endodontic and restorative procedures. *Journal of Endodontia*.

[37] Eakle W. S., Maxwell E. H., Braly B. V. (1986). Fractures of posterior teeth in adults. *The Journal of the American Dental Association*.

[38] Bernardo M., Luis H., Martin M. D. (2007). Survival and reasons for failure of amalgam versus composite posterior restorations placed in a randomized clinical trial. *The Journal of the American Dental Association*.

[39] Soncini J. A., Maserejian N. N., Trachtenberg F., Tavares M., Hayes C. (2007). The longevity of amalgam versus compomer/composite restorations in posterior primary and permanent teeth: findings from the New England Children's Amalgam Trial. *The Journal of the American Dental Association*.

[40] Opdam N. J. M., Bronkhorst E. M., Loomans B. A. C., Huysmans M.-C. D. N. J. M. (2010). 12-year survival of composite vs. amalgam restorations. *Journal of Dental Research*.

[41] Meral E., Baseren N. (2019). Shear bond strength and microleakage of novel glass-ionomer cements: an in vitro study. *Nigerian Journal of Clinical Practice*.

[42] Amesti-Garaizabal A., Agustín-Panadero R., Verdejo-Solá B. (2019). Fracture resistance of partial indirect restorations made with CAD/CAM technology. A systematic review and meta-analysis. *Journal of Clinical Medicine*.

[43] Tunac A. T., Celik E. U., Yasa B. (2019). Two‐year performance of CAD/CAM fabricated resin composite inlay restorations: a randomized controlled clinical trial. *Journal of Esthetic and Restorative Dentistry*.

[44] Emsermann I., Eggmann F., Krastl G., Weiger R., Amato J. (2019). Influence of pretreatment methods on the adhesion of composite and polymer infiltrated ceramic cad-cam blocks. *The Journal of Adhesive Dentistry*.

[45] Simsek H., Derelioglu S. (2016). In vitro comparative analysis of fracture resistance in inlay restoration prepared with CAD-CAM and different systems in the primary teeth. *BioMed Research International*.

[46] Papadopoulos C., Dionysopoulos D., Tolidis K., Kouros P., Koliniotou-Koumpia E., Tsitrou E. A. (2019). Structural integrity evaluation of large MOD restorations fabricated with a bulk-fill and a CAD/CAM resin composite material. *Operative Dentistry*.

[47] Ausiello P., Apicella A., Davidson C. L., Rengo S. (2001). 3D-finite element analyses of cusp movements in a human upper premolar, restored with adhesive resin-based composites. *Journal of Biomechanics*.

[48] Uludag B., Ozturk O., Ozturk A. N. (2009). Microleakage of ceramic inlays luted with different resin cements and dentin adhesives. *The Journal of Prosthetic Dentistry*.

[49] Hikita K., van Meerbeek B., de Munck J. (2007). Bonding effectiveness of adhesive luting agents to enamel and dentin. *Dental Materials*.

[50] Pouyanfar H., Seyed Tabaii E., Aghazadeh S., Tabatabaei Navaei Nobari S. P., Imani M. M. (2018). Microtensile bond strength of composite to enamel using universal adhesive with/without acid etching compared to etch and rinse and self-etch bonding agents. *Open Access Macedonian Journal of Medical Sciences*.

